# Invasion of the Brain by *Listeria monocytogenes* Is Mediated by InlF and Host Cell Vimentin

**DOI:** 10.1128/mBio.00160-18

**Published:** 2018-02-27

**Authors:** Pallab Ghosh, Elizabeth M. Halvorsen, Dustin A. Ammendolia, Nirit Mor-Vaknin, Mary X. D. O’Riordan, John H. Brumell, David M. Markovitz, Darren E. Higgins

**Affiliations:** a Department of Microbiology and Immunobiology, Harvard Medical School, Boston, Massachusetts, USA; b Cell Biology Program and SickKids IBD Centre, Hospital for Sick Children, Toronto, Ontario, Canada; c Department of Molecular Genetics and Institute of Medical Science, University of Toronto, Toronto, Ontario, Canada; d Division of Infectious Diseases, Department of Internal Medicine, University of Michigan Medical Center, Ann Arbor, Michigan, USA; e Department of Microbiology and Immunology, University of Michigan Medical School, Ann Arbor, Michigan, USA; University of Illinois at Chicago

**Keywords:** InlF, *Listeria monocytogenes*, adhesion, brain, invasion, meningitis, vimentin

## Abstract

*Listeria monocytogenes* is a facultative intracellular bacterial pathogen that is frequently associated with food-borne infection. Of particular concern is the ability of *L. monocytogenes* to breach the blood-brain barrier, leading to life-threatening meningitis and encephalitis. The mechanisms used by bacterial pathogens to infect the brain are not fully understood. Here we show that *L. monocytogenes* is able to utilize vimentin for invasion of host cells. Vimentin is a type III intermediate filament protein within the cytosol but is also expressed on the host cell surface. We found that *L. monocytogenes* interaction with surface-localized vimentin promoted bacterial uptake. Furthermore, in the absence of vimentin, *L. monocytogenes* colonization of the brain was severely compromised in mice. The *L. monocytogenes* virulence factor InlF was found to bind vimentin and was necessary for optimal bacterial colonization of the brain. These studies reveal a novel receptor-ligand interaction that enhances infection of the brain by *L. monocytogenes* and highlights the importance of surface vimentin in host-pathogen interactions.

## IMPORTANCE

*Listeria monocytogenes* is an intracellular bacterial pathogen that is capable of invading numerous host cells during infection. *L. monocytogenes* can cross the blood-brain barrier, leading to life-threatening meningitis. Here we show that an *L. monocytogenes* surface protein, InlF, is necessary for optimal colonization of the brain in mice. Furthermore, in the absence of vimentin, a cytosolic intermediate filament protein that is also present on the surface of brain endothelial cells, colonization of the brain was significantly impaired. We further show that InlF binds vimentin to mediate invasion of host cells. This work identifies InlF as a bacterial surface protein with specific relevance for infection of the brain and underscores the significance of host cell surface vimentin interactions in microbial pathogenesis.

## INTRODUCTION

*Listeria monocytogenes* is one of a select group of bacterial pathogens, including *Haemophilus influenzae*, *Neisseria meningitidis*, *Escherichia coli*, and *Streptococcus pneumoniae*, that are able to invade the brain to cause life-threatening meningitis (1–4). Bacterial meningitis is typically severe, and while most people who receive treatment recover, infections can cause serious complications such as brain damage, hearing loss, or learning disabilities in children. *L. monocytogenes* is predicted to account for at least 10% of all community-acquired meningitis in the United States (5). No vaccine currently exists for *L. monocytogenes*, and the ability of bacteria to effectively invade host cells may uniquely aid in the systemic dissemination necessary to cross the blood-brain barrier (BBB) and colonize the brain.

*L. monocytogenes* is capable of invading numerous nonprofessional phagocytic host cells through the interactions of bacterial surface proteins with host cell surface receptors (6). Two of the best-characterized interactions facilitating *L. monocytogenes* invasion of host cells involve the internalin family proteins InlA and InlB and their host cell receptors E-cadherin and the Met receptor, respectively (7, 8). Nonetheless, a role for the majority of the >25 internalin family members has yet to be determined (8–11). Prior studies have shown that InlB is required for invasion of cultured human brain microvascular endothelial cells (HBMEC), while deletion of *inlA* had no effect on HBMEC infection (12, 13). However, InlA and InlB do not appear to play a role in direct infection of the brain *in vivo* (14–17), suggesting that E-cadherin and the Met receptor may not contribute to penetration of the BBB. Thus, the identity of specific factors necessary to facilitate *L. monocytogenes* infection of the brain has remained unclear.

Here, we report that a member of the internalin family of surface proteins, InlF, plays a role in *L. monocytogenes* colonization of the brain *in vivo*. Previous *L. monocytogenes* infection studies using mice did not reveal a general virulence defect of an *inlF* deletion mutant (Δ*inlF*) in systemic dissemination and colonization of the liver and spleen (9, 18). Here, we have determined that InlF is necessary for efficient colonization of the brain during *in vivo* infection in mice. Additionally, we have shown biochemically, by using affinity chromatography/mass spectrometry and immunoprecipitation analyses, that purified InlF can interact with host cell vimentin. Using immunofluorescence confocal microscopy, we have shown that InlF-expressing *L. monocytogenes* binds to cell surface vimentin to mediate adhesion of mammalian brain endothelial cells. Furthermore, during *in vivo* infection, *L. monocytogenes* is deficient in colonization of the brains of vimentin knockout mice. To our knowledge, these studies represent the first reported interaction of a host cell receptor and an *L. monocytogenes* surface protein with specific relevance to colonization of the brain during infection.

## RESULTS

### InlF mediates *L. monocytogenes* invasion of the brain *in vivo.*

**FIG 1:**
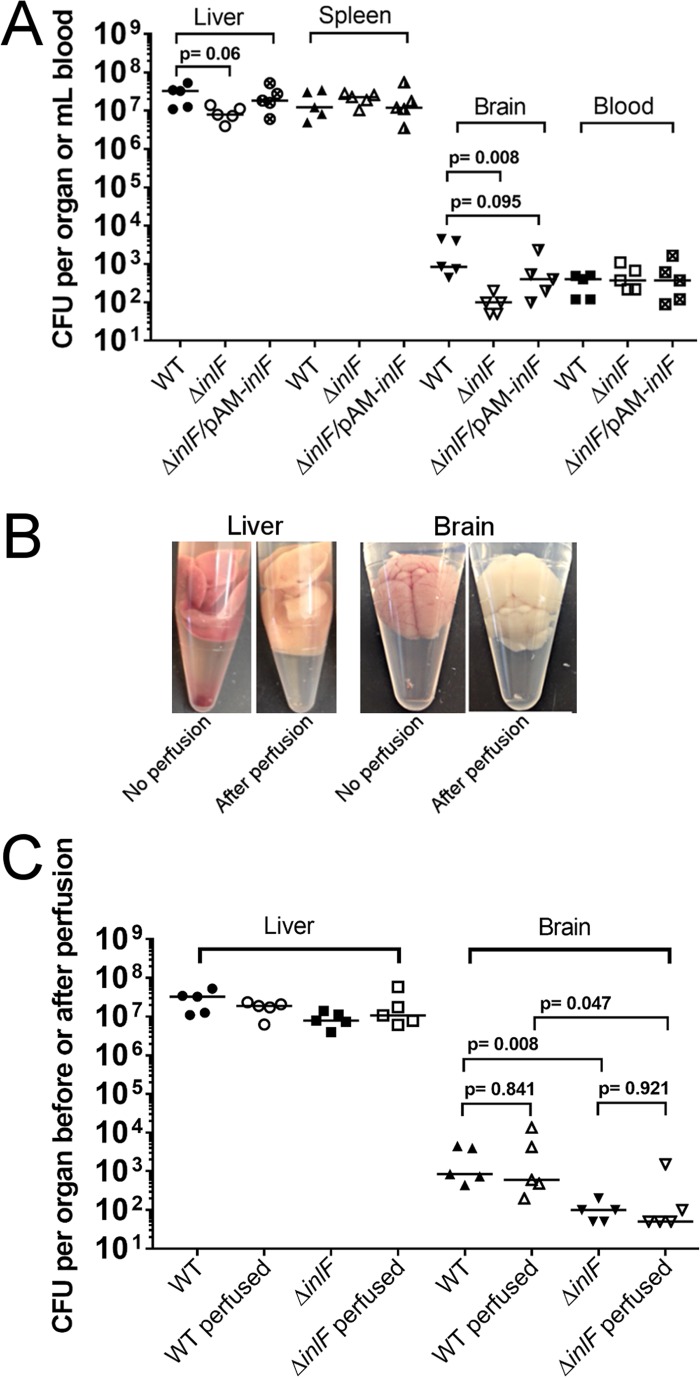
Requirement of InlF for *L. monocytogenes* infection *in vivo*. BALB/c mice were infected intravenously with *L. monocytogenes* 10403S wild-type (WT), Δ*inlF* mutant, or Δ*inlF/*pAM-*inlF* mutant bacteria (1 × 10^4^ to 2 × 10^4^/animal). (A) At 72 h postinfection, the liver, spleen, brain, and blood of each mouse were collected and the bacterial burden was determined. (B) At 72 h postinfection, the liver and brain were collected or euthanized mice were perfused through the heart with 20 ml of PBS containing 10 mM EDTA. Representative organs from nonperfused and perfused mice are shown. (C) At 72 h postinfection, the bacterial burden in the liver and brain was determined as described for panel A or mice were perfused as described for panel B and the bacterial burden in the liver and brain was determined. Horizontal lines indicate median values. *P* represents the statistical significance of the difference between bracketed groups.

We hypothesized that InlF may play a role in *L. monocytogenes* dissemination and the colonization of specific organs during *in vivo* infection. To test this hypothesis, we infected mice by intravenous injection of wild-type *L. monocytogenes* 10403S or an isogenic deletion mutant lacking InlF (Δ*inlF*). The number of bacteria present in the liver, spleen, and brain of each mouse was determined 72 h postinfection. We discovered that InlF is necessary for efficient colonization of the brain (Fig. 1A). The bacterial burden was reduced by ~1 log specifically in the brains of mice infected with the Δ*inlF* mutant. This defect could be complemented when InlF was expressed in *trans* from a plasmid in the Δ*inlF* mutant strain (Δ*inlF*/pAM-*inlF*) (Fig. 1A). In contrast to colonization of the brain, InlF did not have a significant role in colonization of the other organs examined. These findings reveal that InlF has a specific role in colonization of the brain by *L. monocytogenes*.

The brain is highly vascularized, and *L. monocytogenes* is known to infect cell types present in the blood (1, 19). Therefore, it was conceivable that the observed role for InlF in colonization of the brain was due to a necessity of InlF for colonization of the blood. To test this possibility, we examined the bacterial burden in the blood of infected mice. We observed no difference in bacterial numbers in the blood between the strains examined (Fig. 1A). In separate experiments, we performed whole-body perfusion of mice to remove blood from organs (Fig. 1B). Perfusion of animals did not significantly affect the bacterial burden in the brain or liver (Fig. 1C). Thus, the defect in colonization of the brain by the Δ*inlF* mutant was not due to a defect in the ability of Δ*inlF* mutant bacteria to colonize the blood.

Histopathological analyses of brain tissue from mice infected with wild-type bacteria revealed inflammation of the lateral ventricles (see Fig. S1B in the supplemental material) characteristic of *L. monocytogenes* meningoencephalitis (20). These histological changes were not observed in the brains of uninfected control animals or in Δ*inlF* mutant-infected mice (Fig. S1A and C). Gram-positive bacteria were detected in the periventricular brain stem parenchyma of wild-type-infected mice (Fig. S1B). Overall, these findings indicate that InlF plays a significant role in *L. monocytogenes* infection of the brain during systemic infection.

10.1128/mBio.00160-18.2 FIG S1  Histological analysis of the brains of mice infected with *L. monocytogenes*. BALB/c mice were infected intravenously with 10403S wild-type (WT) or Δ*inlF* mutant bacteria at 1 × 10^4^ to 2 × 10^4^/animal. At 72 h postinfection, mice were euthanized; the brains were removed from their skulls, fixed in Bouin’s fluid (Electron Microscopy Sciences, Hatfield, PA), and embedded in paraffin; and 5-µm progressive sections were stained with either hematoxylin and eosin or Gram stain. (A) Hematoxylin-and-eosin-stained brain (coronal section) of an uninfected control mouse (×200 magnification; scale bar = 200 µm). A lateral ventricular space is shown in the boxed area and magnified in the upper right inset. (B) Hematoxylin-and-eosin-stained brain (coronal section) of a wild-type-infected mouse showing acute inflammation in the lateral ventricular spaces (shown in the boxed area and magnified in the upper right inset). Gram-stained bacteria in the boxed area are shown magnified in the lower right inset. Arrows indicate rod-shaped Gram-positive bacteria. (C) Hematoxylin-and-eosin-stained brain (sagittal section) of a Δ*inlF* mutant-infected mouse. Acute inflammation in ventricular spaces was not observed (shown in the boxed area and magnified in the upper right inset). Download FIG S1, TIF file, 9.6 MB. Copyright © 2018 Ghosh et al. 2018 Ghosh et al. This content is distributed under the terms of the Creative Commons Attribution 4.0 International license.

### InlF binds host cell vimentin.

To identify the host cell protein(s) that interacts with InlF, affinity chromatography was performed. Purified InlF protein bound to an affinity matrix was incubated with lysates of L2 cells treated with the ROCK inhibitor Y27632, a condition known to promote InlF-mediated bacterial invasion (18). Host proteins that bound immobilized InlF were eluted and subjected to mass spectrometry analysis. We identified three potential host cell proteins that interact with InlF: vimentin, Sfpq splicing factor, and AHNAK nucleoprotein (Table S1). Of these proteins, vimentin, a type III intermediate filament protein, had the highest sequence coverage at 52.1%. Vimentin is broadly expressed in mesenchymal cells and regulates cell adhesion, transcellular migration, and cellular signaling (21). Interestingly, vimentin is also expressed on the surface of various brain cells (e.g., brain microvascular endothelial cells and astrocytes) (22). Apart from a role for vimentin in the maintenance of cytoskeletal architecture, recent studies have suggested that viruses are capable of interacting with vimentin as a component of the cellular adherence mechanism (23). Bacteria such as *E. coli* K1 and group A streptococci also use vimentin as a ligand for host cell attachment to mediate pathogen entry into host cells (24, 25).

10.1128/mBio.00160-18.5 TABLE S1  Mass spectrometry of InlF-interacting partners. The InlF-interacting proteins identified are ranked by the protein sequence coverage found. Download TABLE S1, DOCX file, 0.02 MB. Copyright © 2018 Ghosh et al. 2018 Ghosh et al. This content is distributed under the terms of the Creative Commons Attribution 4.0 International license.

To confirm the interaction of vimentin with InlF, we performed an immunoprecipitation assay with purified, His_6_-tagged InlF and mCherry (red fluorescent protein [RFP])-vimentin from host cell extracts (Fig. S2). Western blot analysis indicated that, contrary to the immobilized RFP control, a significantly larger amount of InlF was recovered following incubation with mCherry-vimentin, demonstrating an InlF-vimentin interaction. The difference in InlF recovery was not attributed to a lower transfection efficiency of the RFP control vector, as the ratio of anti-His to anti-RFP signals determined by densitometry was >5-fold higher in the mCherry-vimentin sample (Fig. S2). Collectively, these studies suggest an important role for vimentin as a conserved host receptor for pathogen adhesion and internalization. Therefore, the role of vimentin was further examined to determine its importance for *L. monocytogenes* invasion of host cells.

10.1128/mBio.00160-18.3 FIG S2  Immunoprecipitation analysis of InlF-vimentin interaction. mCherry-tagged human vimentin or RFP-overexpressing bEnd.3 cell extracts were immunoprecipitated with RFP-trap beads and incubated with purified InlF-His_6_. Western blot analysis of the sample eluates with antibodies against His_6_ (α-His) or RFP (α-RFP) is shown. The optical density ratio of anti-His to anti-RFP signals from each eluate sample was determined by densitometry. Download FIG S2, TIF file, 0.9 MB. Copyright © 2018 Ghosh et al. 2018 Ghosh et al. This content is distributed under the terms of the Creative Commons Attribution 4.0 International license.

### *L. monocytogenes* invasion of host cells is mediated by vimentin.

Withaferin A (WFA) is a natural steroidal lactone that binds vimentin and can function as an inhibitor of vimentin activity. WFA treatment of mammalian cells leads to cleavage of vimentin and reorganization of vimentin intermediate filaments (24–27). Prior studies have shown that HBMEC treated with WFA blocked the invasion of *E. coli* K1, suggesting that vimentin is required for *E. coli* K1 invasion (24). To examine whether *L. monocytogenes* uptake by nonprofessional phagocytic cells is dependent on vimentin, we performed *L. monocytogenes* invasion studies by using gentamicin protection assays with L2 and Neuro-2a cells treated with WFA. As shown in Fig. S3A and B, treatment of host cells with WFA decreased *L. monocytogenes* invasion in a dose-dependent manner. In addition, we tested if there was any effect of WFA on bacterial viability and growth *in vitro*. No difference in the growth of wild-type *L. monocytogenes* was observed during exposure to WFA (5 or 10 µM) compared to a nontreated control culture (Fig. S3C). These data suggest that vimentin is involved in *L. monocytogenes* invasion of host cells.

10.1128/mBio.00160-18.4 FIG S3  Effect of WFA treatment on *L. monocytogenes* invasion of host cells *in vitro*. (A, B) Inhibition of wild-type *L. monocytogenes* strain 10403S invasion of host cells by WFA treatment. L2 (A) and Neuro-2a (B) cells were treated with dimethyl sulfoxide (DMSO) or increasing concentrations of WFA prior to infection with *L. monocytogenes* 10403S for 1 h. Intracellular bacteria were quantified by gentamicin protection assay. (C) Effect of WFA on *L. monocytogenes* 10403S survival and growth in BHI broth. Data in panels A and B represent the mean number of CFU per well ± the standard deviation in one of three experiments performed in triplicate with similar results. **, *P* < 0.01; ***, *P* < 0.001. Data in panel C represent the mean number of CFU per milliliter ± the standard deviation in one of two experiments performed in triplicate with similar results. Download FIG S3, TIF file, 0.1 MB. Copyright © 2018 Ghosh et al. 2018 Ghosh et al. This content is distributed under the terms of the Creative Commons Attribution 4.0 International license.

**FIG 2:**
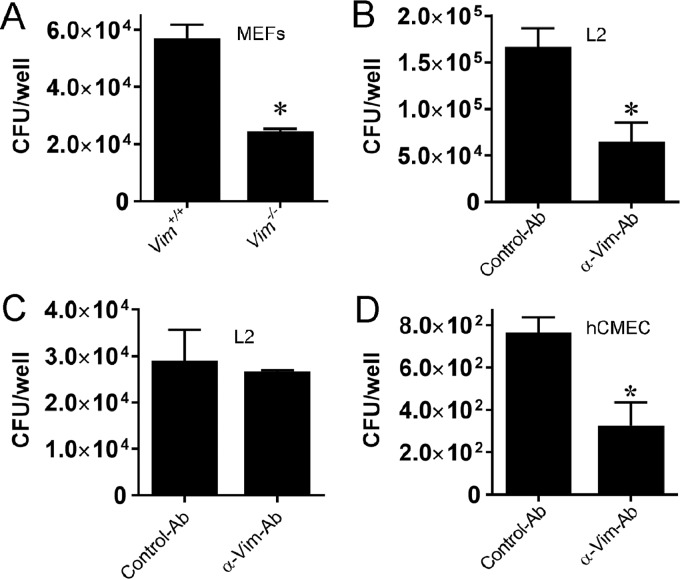
Vimentin mediates *L. monocytogenes* invasion of host cells. (A) Comparison of *L. monocytogenes* invasion in host cells expressing or lacking vimentin. MFT-6 (Vim^+/+^) or MFT-16 (Vim^−/−^) mouse embryo fibroblasts (MEFs) were infected with wild-type *L. monocytogenes* 10403S for 1 h. Intracellular bacteria were quantified by gentamicin protection assay. (B to D) Inhibition of *L. monocytogenes* invasion of host cells following treatment with anti-vimentin antibody (α-Vim Ab). L2 cells (B, C) or hCMEC (D) were incubated for 1 h with 40 μg of anti-vimentin polyclonal antibody or an isotype control antibody prior to infection with wild-type *L. monocytogenes* 10403S (B and D) or Δ*inlF* mutant (C) bacteria for gentamicin protection assays. Data represent the mean ± standard deviation number of CFU per well in one of three experiments performed in triplicate with similar results. *, *P* < 0.05.

Several studies have suggested that bacterial pathogens use vimentin as a receptor for host cell adherence to mediate pathogen entry into host cells or to cross host barriers (24, 25, 28–30). To examine the importance of vimentin for *L. monocytogenes* invasion *in vitro*, we performed gentamicin protection assays with MFT-6 (Vim^+/+^) and MFT-16 (Vim^−/−^) mouse embryo fibroblasts (31). As shown in Fig. 2A, infection of MFT-16 cells, which lack vimentin, resulted in a >2-fold decrease in intracellular bacteria compared to infection of vimentin-expressing MFT-6 cells. To investigate if *L. monocytogenes* utilizes vimentin as a receptor for host cell invasion, we initially determined whether *L. monocytogenes* invasion could be inhibited by blocking surface vimentin. Anti-vimentin polyclonal antibody was incubated with L2 fibroblasts to prevent bacterial binding to cell surface vimentin. Anti-vimentin antibody pretreatment significantly reduced (3-fold) wild-type *L. monocytogenes* invasion of L2 cells compared to treatment with an isotype control antibody (Fig. 2B). Moreover, in contrast to the observed 3-fold reduction of invasion in L2 cells pretreated with anti-vimentin antibody by wild-type bacteria, no reduction in bacterial invasion was observed in L2 cells pretreated with anti-vimentin antibody and infected with Δ*inlF* mutant bacteria (Fig. 2C). Importantly, to determine if vimentin is important for bacterial invasion of endothelial cells relevant to brain infection, we determined whether *L. monocytogenes* invasion of human cerebral microvascular endothelial cells (hCMEC) (32) would be inhibited by blocking surface vimentin. Anti-vimentin antibody pretreatment also significantly reduced (~3-fold) wild-type *L. monocytogenes* invasion of hCMEC compared to treatment with the isotype control antibody (Fig. 2D). Collectively, these results demonstrate a role for surface vimentin in *L. monocytogenes* invasion of host cells, including cell types relevant to infection of the BBB and brain, and suggest that InlF mediates invasion of host cells through an interaction with surface-localized vimentin.

### InlF facilitates *L. monocytogenes* association with host cell surface vimentin.

**FIG 3:**
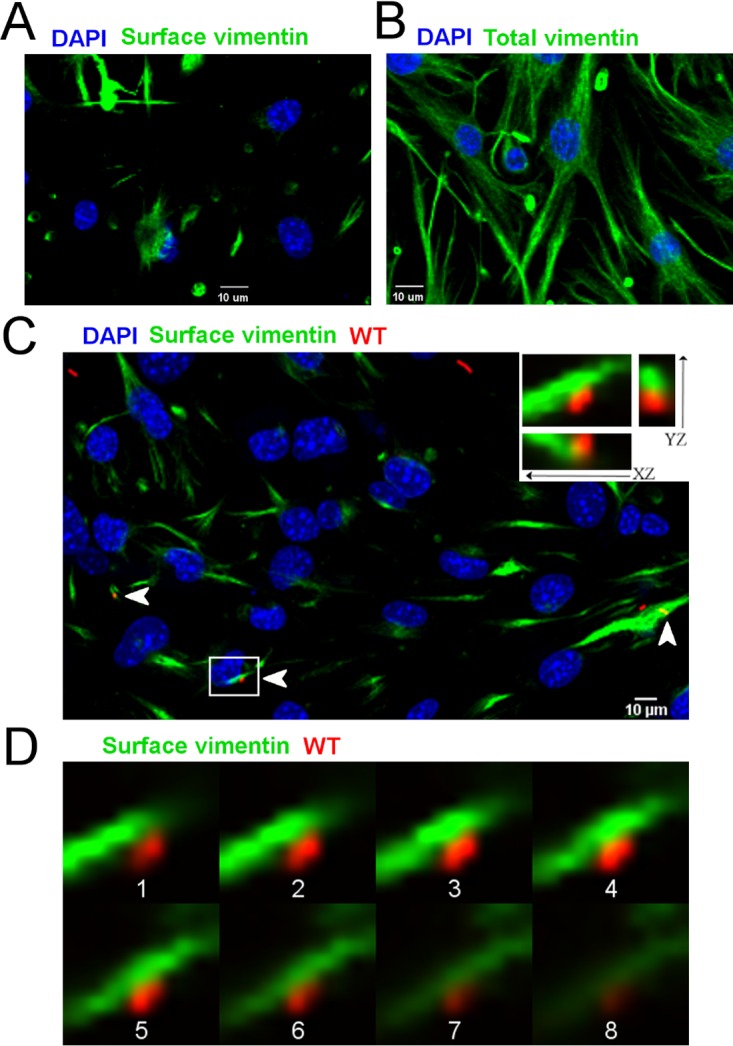
*L. monocytogenes* interacts with cell surface vimentin. (A) Expression of cell surface vimentin on nonpermeabilized bEnd.3 cells. Host cell monolayers were treated with CytoD and fixed with 3% paraformaldehyde. Nonpermeabilized, fixed bEnd.3 cells were then stained with anti-vimentin polyclonal antibody (green), and host cell nuclei were counterstained with 4',6-diamidino-2-phenylindole (DAPI) (blue). (B) Expression of total (cytosolic and surface) vimentin in permeabilized bEnd.3 cells. Vimentin was detected with anti-vimentin polyclonal antibody, and host cell nuclei were counterstained with DAPI as described for panel A. (C) CytoD-treated bEnd.3 cells were infected at a multiplicity of infection of 25 with wild-type (WT) *L. monocytogenes* 10403S for 2 h. Nonpermeabilized, fixed cells were then stained with antibodies against surface vimentin (green) and *L. monocytogenes* (red). Nuclei were counterstained with DAPI (blue). Samples were then analyzed by confocal microscopy. A representative confocal *z*-stack is shown. Arrows indicate association between bEnd.3 cell surface vimentin and *L. monocytogenes*. Inset images are higher magnifications of the boxed area. The orthogonal projections of the optical section were viewed from the *xz* and *yz* planes. (D) Confocal microscopy *z* planes (1 to 8) from the boxed area in panel C acquired from the basal side of the bEnd.3 cell to the apical side (bottom to top). The thickness of each plane was 0.3 µm. Scale bar = 10 µm.

Vimentin is a cytoplasmic intermediate filament protein (33). However, multiple studies indicate that vimentin can also be present on the surface of numerous cell types, including skeletal muscle cells, activated macrophages, vascular endothelial cells, and brain cells (22, 24, 25, 34, 35). Indeed, in agreement with these prior studies, we observed robust expression of vimentin on the surface of bEnd.3 mouse brain endothelial cells, as detected by immunofluorescence staining of nonpermeabilized cells (Fig. 3A and B). We hypothesized that *L. monocytogenes* associates with surface-expressed vimentin to promote subsequent invasion of host cells. To test this hypothesis, we pretreated bEnd.3 cells with cytochalasin D (CytoD) prior to infection. CytoD treatment prevents actin-mediated entry of *L. monocytogenes* into host cells (7) but does not affect the expression of host cell vimentin (36, 37). CytoD-treated bEnd.3 cells were incubated with wild-type *L. monocytogenes* 10403S, and confocal immunofluorescence microscopy experiments were performed to visualize the surface localization of *L. monocytogenes* and host cell surface vimentin (Fig. 3C and D). Colocalization of bacteria with vimentin on the surface of bEnd.3 cells was observed, consistent with a role for vimentin in promoting the uptake of *L. monocytogenes* by host cells (Fig. 3C and D).

**FIG 4:**
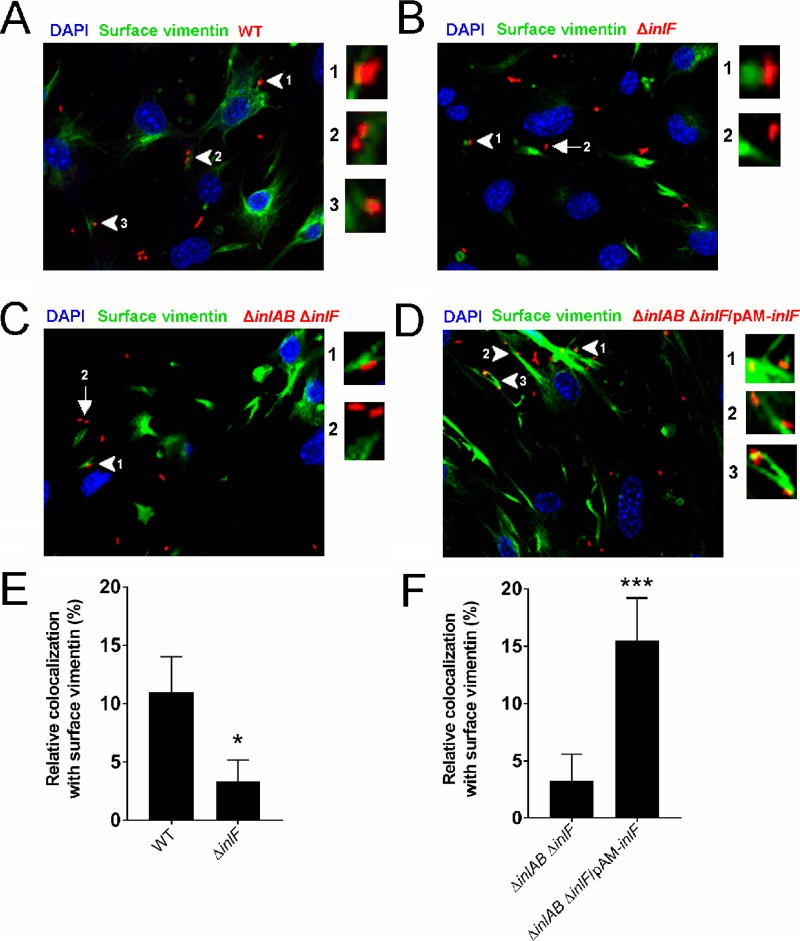
Requirement of InlF for *L. monocytogenes* interaction with cell surface vimentin. CytoD-treated bEnd.3 cells were infected at an MOI of 25 with *L. monocytogenes* 10403S wild-type (WT) (A), Δ*inlF* mutant (B), Δ*inlAB* Δ*inlF* mutant (C), or Δ*inlAB* Δ*inlF*/pAM-*inlF* mutant (D) bacteria for 2 h. The nonpermeabilized, fixed cells were then immunostained as described in the legend to Fig. 3. Representative confocal *z*-stacks are shown. Arrowheads magnified to the right indicate bEnd.3 cell surface vimentin colocalization with wild-type, Δ*inlF* mutant, Δ*inlAB* Δ*inlF* mutant, or Δ*inlAB* Δ*inlF*/pAM-*inlF* mutant bacteria. Arrows indicate Δ*inlF* or Δ*inlAB* Δ*inlF* mutant bacteria that are not associated with cell surface vimentin. (E, F) Quantification of vimentin colocalization with wild-type and Δ*inlF* mutant bacteria (E) or Δ*inlAB* Δ*inlF* and Δ*inlAB* Δ*inlF*/pAM-*inlF* mutant bacteria (F). Colocalization of surface vimentin with *L. monocytogenes* was assessed from 10 to 15 different confocal image fields examining >400 bacteria of each strain. Bars represent the mean ± the standard error of the mean. *, *P* < 0.05; ***, *P* < 0.001.

Next, we examined the role of InlF in the targeting of *L. monocytogenes* to host cell surface vimentin. We observed that association of the Δ*inlF* mutant with cell surface vimentin was significantly reduced compared to that of wild-type bacteria (Fig. 4A, B, and E). These findings indicate that InlF mediates the association of *L. monocytogenes* with host cells via cell surface vimentin. It is known that *L. monocytogenes* can invade host cells via InlA and InlB, which bind to their host cell receptors E-cadherin and the Met receptor, respectively (7, 8). It was conceivable that InlF binding to vimentin was facilitated by interaction of InlA or InlB with host cell surface receptors. To test this possibility, we generated a triple knockout strain lacking all three internalins (Δ*inlAB* Δ*inlF*). Very little association of Δ*inlAB* Δ*inlF* mutant bacteria with cell surface vimentin was observed (Fig. 4C and F). However, complementation of the Δ*inlAB* Δ*inlF* mutant by expression of InlF from a plasmid (Δ*inlAB* Δ*inlF*/pAM-*inlF*) led to ~4-fold greater colocalization with vimentin compared to that of the Δ*inlAB* Δ*inlF* mutant (Fig. 4D and F). Thus, InlF can mediate bacterial association with host cell surface vimentin independently of InlA and InlB. Taken together, these results further indicate that InlF mediates the adherence of host cells through an interaction with surface-localized vimentin.

### Vimentin is required for efficient invasion of the brain by *L. monocytogenes in vivo.*

**FIG 5:**
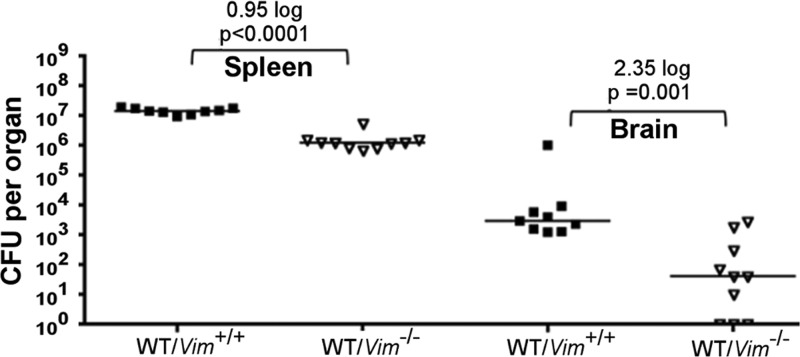
Requirement of vimentin for *in vivo* virulence of *L. monocytogenes*. Vimentin knockout (Vim^−/−^) and control (Vim^+/+^) mice were infected intravenously with wild-type (WT) *L. monocytogenes* 10403S bacteria (1 × 10^5^/animal). At 48 h postinfection, the spleen and brain of each mouse were collected and the bacterial burden was determined. Horizontal lines indicate median values. The log_10_ reduction values are indicated. *P* represents the statistical significance of the difference between bracketed groups.

Vimentin has been shown to be present on the surface of various cell types in the brain, including brain microvascular endothelial cells (22, 24). Therefore, we investigated the impact of vimentin on *L. monocytogenes* infection of the brain *in vivo*. Vimentin knockout mice (38) were infected by intravenous injection of wild-type 10403S bacteria. The number of bacteria present in the spleen and brain of each mouse was determined 48 h postinfection. Vimentin knockout mice infected with wild-type *L. monocytogenes* showed a >2-log decrease in colonization of the brain compared to the bacterial burden in control mice (Fig. 5). There was also a significant (~1-log) decrease in colonization of the spleen in vimentin knockout mice compared to that in control mice. These data demonstrate that vimentin is important for *L. monocytogenes* infection *in vivo* and in particular for successful colonization of the brain.

## DISCUSSION

The identities of specific factors necessary to facilitate bacterial infection of the brain *in vivo* have remained unclear. Here we report the discovery that both vimentin and InlF are required for efficient colonization of the brain by *L. monocytogenes*. *In vivo* infection experiments with vimentin knockout mice (Fig. 5) and infection of wild-type mice with Δ*inlF* mutant bacteria (Fig. 1A) suggested that the presence of vimentin and expression of InlF by *L. monocytogenes* are necessary for maximal colonization of the brain in mice. To our knowledge, InlF and vimentin are the first bacterial surface protein and host cell receptor, respectively, to be identified with specific relevance for *L. monocytogenes* infection of the brain *in vivo*. Given our additional *in vitro* data demonstrating vimentin-dependent host cell invasion by *L. monocytogenes* (Fig. 2) and the InlF-dependent colocalization of bacteria with surface-associated vimentin (Fig. 3 and 4), we propose a model in which *L. monocytogenes* uses InlF to directly interact with surface vimentin to penetrate host cells and colonize the brain. Thus, the InlF-vimentin interaction represents a novel step in the pathogenesis of *L. monocytogenes* leading to bacterial meningitis. However, we noticed that the complete inhibition of host cell invasion by *L. monocytogenes* was not achieved when using the vimentin null cells and that confocal immunofluorescence microscopy showed a low level of *L. monocytogenes* interaction with host cell surface vimentin in the absence of InlF. These observations support an additional mechanism(s) of *L. monocytogenes* invasion of host cells besides the InlF-vimentin interaction.

While *L. monocytogenes* encodes >25 internalin family members, a role for the majority of these determinants in either tissue- or species-specific pathogenesis has not been shown (39). The well-studied *L. monocytogenes* invasion proteins InlA and InlB do not appear to play a significant role in direct infection of the brain *in vivo* (14–16). Recently, a previously uncharacterized internalin family member, InlP, was shown to play an important role in *L. monocytogenes* infection of the placenta in guinea pigs and mice (40). Expression of InlP caused a 3-log increase in the bacterial burden in the placenta while having a minor effect on the colonization of other maternal organs. Our identification of InlF as a novel virulence factor for colonization of the brain strengthens a model in which *L. monocytogenes* encodes numerous internalin family members to facilitate tissue-specific invasion of host cells through interactions with host cell-specific receptors. The InlF protein has many features characteristic of internalin proteins, such as a signal sequence, two repeat regions, an LPXTG motif, and a C-terminal cell wall anchor. However, the *inlF* gene is neither located in an operon nor controlled by the known *L. monocytogenes* regulators PrfA, σ^B^, or VirR (9, 41, 42). The *inlF* gene is conserved among the most common pathogenic lineages of *L. monocytogenes*, including all sequenced lineage II strains of *L. monocytogenes*, and has 75 to 80% sequence identity to the lineage I strains (43).

Vimentin has historically been viewed as a cytosolic intermediate filament protein that forms static cytoskeletal networks important for cell structural integrity (44). However, numerous studies have now shown that vimentin plays a more dynamic function in multiple cellular processes, including autophagy, cell adhesion, and innate immune signaling (33). Many of these cellular functions are important for host-pathogen interactions during bacterial infections. Indeed, an increasing number of reports have demonstrated a diverse role for vimentin in bacterial infections, primarily in innate host cell defense mechanisms and pathogen adhesion and invasion (45). Vimentin has been shown to be an important invasion receptor for the IbeA protein of meningitic *E. coli* K1 and to facilitate invasion of the brain by *E. coli* K1 *in vivo* (24, 28, 46). We have shown for the first time that vimentin is exploited by *L. monocytogenes* for invasion and colonization of the brain *in vivo*. In addition, we have shown that *L. monocytogenes* InlF facilitates bacterial association with surface vimentin and mediates colonization of the brain *in vivo*. Our studies, along with others, may indicate that vimentin is a central meningitic factor utilized by multiple bacterial pathogens to facilitate crossing of the BBB and colonization of the brain. Because *L. monocytogenes* is a model organism for elucidating the mechanisms of intracellular pathogenesis and invasion of the central nervous system, a greater understanding of InlF-vimentin interactions may prove highly applicable to other pathogens and provide significant insight and possible targets for the development of novel therapeutics for meningitic infections.

## MATERIALS AND METHODS

For a detailed description of the materials and methods used in this study, see Text S1 in the supplemental material.

10.1128/mBio.00160-18.1 TEXT S1  Supplemental materials and methods used in this study. Download TEXT S1, DOCX file, 0.04 MB. Copyright © 2018 Ghosh et al. 2018 Ghosh et al. This content is distributed under the terms of the Creative Commons Attribution 4.0 International license.

### Bacterial strains and media.

*L. monocytogenes* strains were grown in brain heart infusion (BHI) medium (Difco, Detroit, MI). Chloramphenicol was used at 7.5 μg/ml for selection of plasmids pAM401spacOid-BamHI, pAM-*inlF*, and pAM-*inlF*-His in *L. monocytogenes* (18).

### InlF-His_6_ protein expression and purification.

The *inlF* gene was cloned into plasmid pAM401spacOid-BamHI (18). To express InlF-His_6_, the resulting plasmid, pAM-*inlF*-His, was introduced into wild-type *L. monocytogenes* 10403S by electroporation to generate strain DH-L1899. DH-L1899 was grown for 15 h at 37°C in BHI medium containing 7.5 μg/ml chloramphenicol. The DH-L1899 culture was pelleted, the supernatant was supplemented with 10 mM imidazole, and the pH was adjusted to 8.0. The supernatant was then filtered through a 0.2-μm filter flask (Millipore, Billerica, MA). The filtered supernatant containing secreted InlF-His_6_ was cycled over Ni-nitrilotriacetic acid (NTA) resin (Qiagen, Valencia, CA) at 4°C. The Ni-NTA column was washed with cold wash buffer (50 mM NaH_2_PO_4_, 300 mM NaCl, 10 mM imidazole, pH 8.0), and InlF-His_6_ was eluted off the column in cold elution buffer (50 mM NaH_2_PO_4_, 300 mM NaCl, 250 mM imidazole, pH 8.0).

### Tissue culture extraction.

L2 cells were grown in RPMI 1640 medium as described in Text S1. To harvest L2 cells, cold NP-40 buffer (phosphate-buffered saline [PBS], pH 7.5, 150 mM NaCl, 1% NP-40) containing protease inhibitor cocktail (Sigma, St. Louis, MO) was added and cells were detached with a cell scraper and transferred into a 15-ml tube. Cells were lysed at 4°C for 30 min with end-over-end rotation. The cell lysate was centrifuged at 4°C at 1,000 × *g* for 10 min, and the supernatant was collected for use in affinity chromatography.

### Affinity chromatography.

The Affi-Gel 15 affinity support system (Bio-Rad, Hercules, CA) was used for affinity chromatography, and samples were prepared in accordance with the manufacturer’s recommendations. Details are provided in Text S1.

### Gentamicin protection assay.

Host cell infection studies were carried out as described previously (18). Detailed procedures are provided in Text S1.

### Confocal fluorescence microscopy.

For immunofluorescence microscopy studies, samples were prepared as described previously (47, 48). Confocal images were acquired with a FluoView FV3000 microscope (Olympus) with a 40× oil immersion objective lens. Details are presented in Text S1.

### *In vivo* virulence studies.

For animal infections with *L. monocytogenes*, female BALB/c mice (6 to 8 weeks of age) were purchased from Jackson Laboratory (Bar Harbor, ME). Vimentin knockout mice were housed at the University of Michigan (49, 50). Mice were injected intravenously with the wild-type 10403S, Δ*inlF* mutant, or Δ*inlF*/pAM-*inlF* mutant strain at 1 × 10^4^ to 2 × 10^4^ or 1 × 10^5^ bacteria/animal. At 48 or 72 h postinfection, mice were humanely euthanized by exposure to CO_2_, followed by cervical dislocation. Blood was collected by cardiac puncture with a 1-ml syringe preloaded with 50 μl of 4% sodium citrate to prevent coagulation. In some experiments, euthanized mice were perfused through the heart with 20 ml of PBS containing 10 mM EDTA. The number of CFU per organ or milliliter of blood was determined by plating dilutions of the blood or organ homogenates. All animal care and experiments were conducted in compliance with the Institutional Animal Care and Use Committee and all federal, state, and local laws.

### Statistical analysis.

Statistical analysis of gentamicin protection assay results was performed with the Student *t* test (two tailed, unpaired). Statistical analysis of *in vivo* virulence study results was performed with the Mann-Whitney U test. Differences were considered significant at *P* < 0.05.
