# Supplementary figures and images for: Invasion of the Brain by Listeria monocytogenes Is Mediated by InlF and Host Cell Vimentin
Source: mBio. 2018 Feb 27;9(1):e00160-18. doi: 10.1128/mBio.00160-18 (PMC5829824; doi:10.1128/mBio.00160-18)

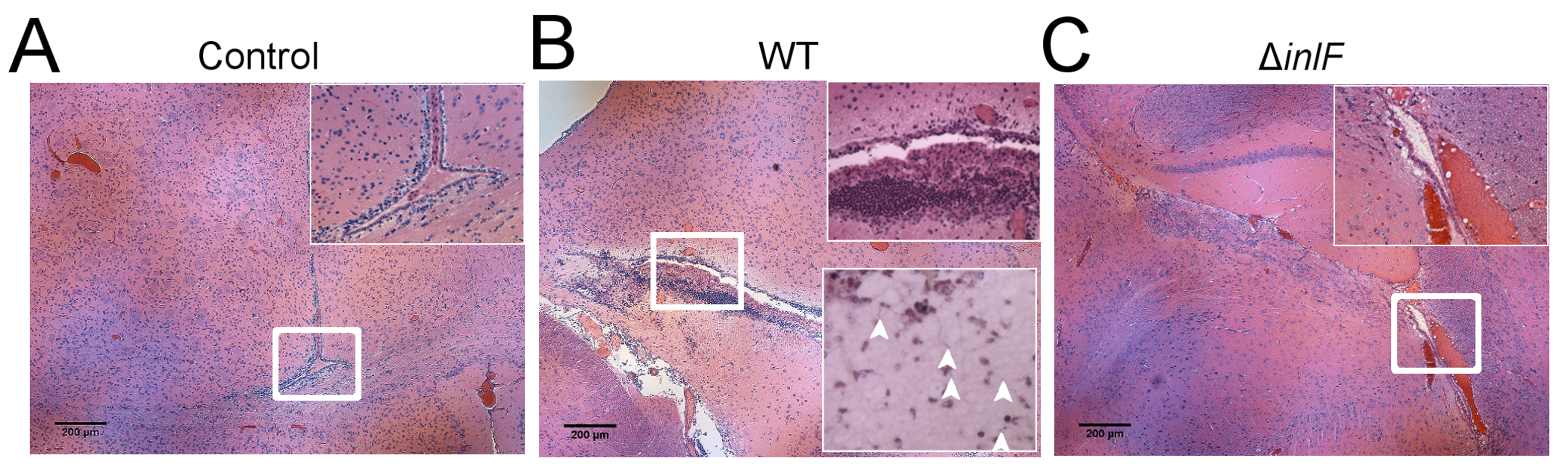

Supplement: FIG S1 [file mbo001183750sf1.tif]

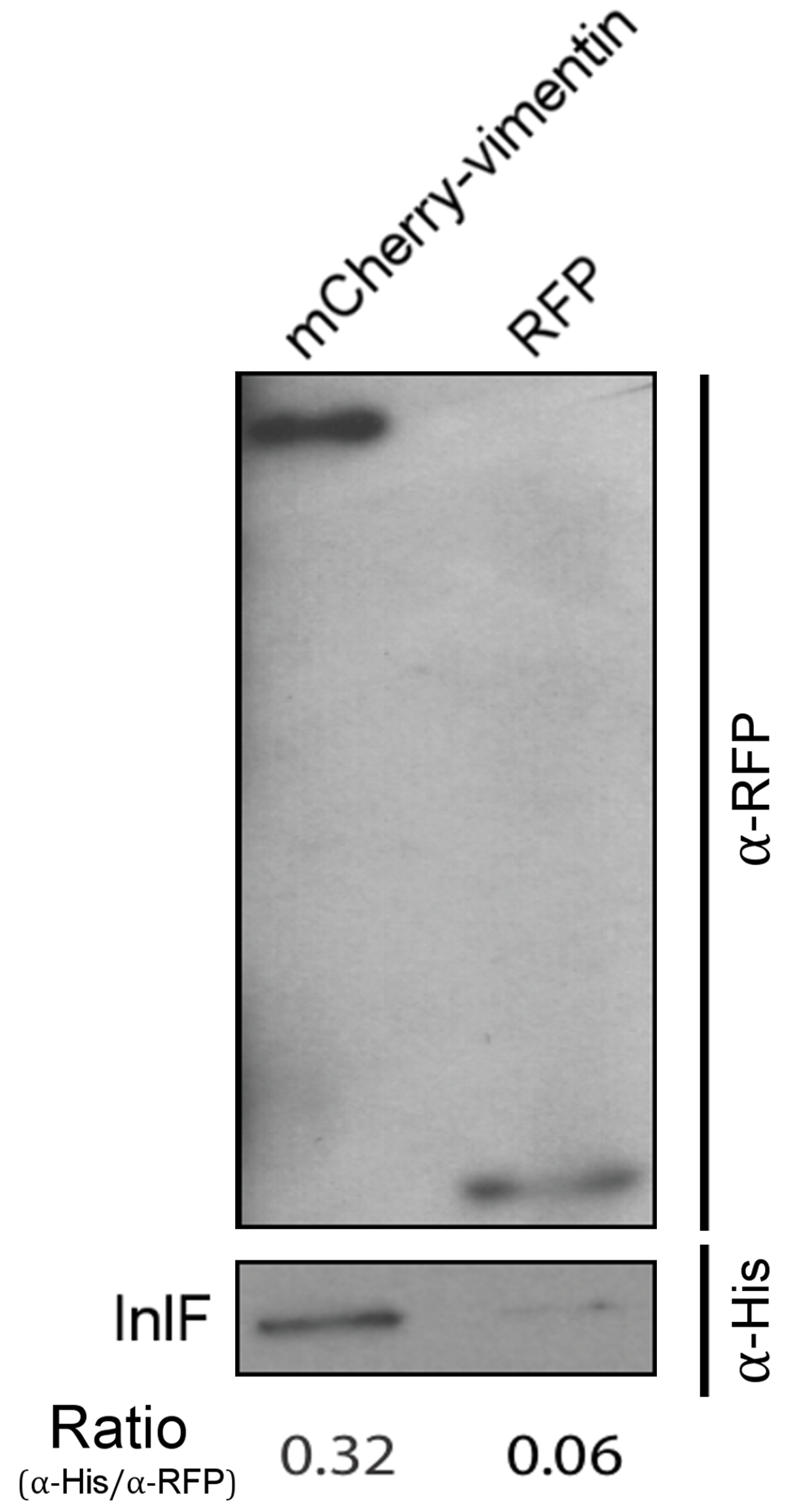

Supplement: FIG S2 [file mbo001183750sf2.tif]
