# Supplementary material for: Invasion of the Brain by Listeria monocytogenes Is Mediated by InlF and Host Cell Vimentin
Source: mBio. 2018 Feb 27;9(1):e00160-18. doi: 10.1128/mBio.00160-18 (PMC5829824; doi:10.1128/mBio.00160-18)
Supplement: TABLE S1 [file mbo001183750st1.docx]

**Table S1.**

Mass spectrometry for InlF-interacting partners. The identified InlF-interacting proteins are ranked by the protein sequence coverage found.

| **Rank order** | **IPI**  **identifier** | **Gene**  **description** | **Gene symbol** | **Sequence coverage (%)*^a^*** | | **Unique peptides*^b^*** | |  |
| --- | --- | --- | --- | --- | --- | --- | --- | --- |
|  |  |  |  |  | |  | |  |
| 1 | IPI00227299.6 | Vimentin | Vim | | 52.1 | | 26 | |
| 2 | IPI00129430.1 | Splicing factor, proline- and glutamine-rich | Sfpq | | 36.2 | | 29 | |
| 3 | IPI00553798.2 | AHNAK nucleoprotein isoform 1 | Ahnak | | 8.3 | | 39 | |

*^a^*Sequence coverage based on amino acid count

*^b^*Uniqueness defined by peptide sequence
