# Supplementary material for: Invasion of the Brain by Listeria monocytogenes Is Mediated by InlF and Host Cell Vimentin
Source: mBio. 2018 Feb 27;9(1):e00160-18. doi: 10.1128/mBio.00160-18 (PMC5829824; doi:10.1128/mBio.00160-18)
Supplement: TEXT S1 [file mbo001183750s1.docx]

**Supplemental Material**

**Supplemental Methods**

*Tissue cell culture*

The mouse brain endothelial cell line (bEnd.3) (ATCC, #CRL-229) was cultured in DMEM medium (Mediatech, Manassas, VA) supplemented with 10% fetal bovine serum, 1 mM sodium pyruvate and 2 mM glutamine. The human cerebral microvascular endothelial cell line (hCMEC) (Cedarlane Labs, Ontario, Canada) was grown in EndoGro™ medium (Millipore, Burlington, MA) according to the manufacturer’s instruction. The murine-derived fibroblast cell line L2 was cultured in RPMI 1640 medium (Mediatech, Manassas, VA) supplemented with 10% fetal bovine serum (HyClone, Logan, UT), 55 μM β-mercaptoethanol, 1 mM sodium pyruvate and 2 mM glutamine. The murine-derived neuroblastoma cell line Neuro-2a (ATCC, #CCL-131) was cultured in DMEM medium as described above. The mouse cell lines MFT-6 (*Vim*^+/+^) and MFT-16 (*Vim*^-/-^), derived from a vimentin knockout mouse, were provided by Dr. Marcia Goldberg ([1](#_ENREF_1)). MFT-6 and MFT-16 cells were cultured in DMEM medium as described above. All cell lines were maintained at 37°C in a 5% CO_2_-air atmosphere.

*DNA construct for InlF expression and purification*

To construct plasmid pAM-*inlF*-His, the *inlF* gene without the LPXTG membrane anchoring motif was amplified by PCR (forward primer: 5’-GGATCCAGGAGGAAAAATATGCATCATCATCATCATCACAAATCTAAAAATAATTATTTCAAAC-3’ and reverse primer: 5’- GATATCTTAGTGGTGGTGGTGGTGGTGTTTTAGCTGTTGCTAGTTCTGTAG-3’). The PCR product was inserted into plasmid pAM401spacOid-BamHI ([2](#_ENREF_2)) digested with BamHI and EcoRV. To express InlF-His_6_, the resulting plasmid, pAM-*inlF*-His, was introduced into wild-type *L. monocytogenes* 10403S by electroporation to generate strain DH-L1899.

*Gentamicin protection assay*

Host cells were seeded in 12-well tissue culture plates and grown to 70-80% confluency. On the day of infection, monolayers were washed twice with phosphate buffered saline (PBS) and pre-incubated with or without inhibitors for 1 hour at 37°C in a 5% CO_2_-air atmosphere. Next, bacteria from 15-hour cultures were added to the monolayers at an MOI of 100:1 in tissue culture media with or without inhibitors and incubated for 1 hour. For blocking invasion experiments, L2 and hCMEC monolayers were incubated with 40 μg chicken anti-vimentin antibody (BioLegend) for 1 hour at 37°C before addition of bacteria to the monolayers at an MOI of 50:1 and 25:1, respectively. Chicken IgY was used as an isotype control. Extracellular bacteria were selectively killed by incubating infected monolayers for 1 hour in culture medium containing 30 μg/mL gentamicin. To quantify bacterial invasion of host cells, the monolayers were washed three times with PBS followed by a 1% Triton X-100 lysis and plating dilutions on agar plates for viable intracellular bacteria.

*Tissue culture extraction*

L2 cells were grown to confluency in a 150 cm^2^ flask in RPMI 1640 medium. The confluent monolayer was treated with 30 μM Y27632 for 2 hours at 37°C. The medium was then aspirated off and cells were washed twice with 10 mL cold PBS. To harvest cells, 5 mL of cold NP-40 buffer (PBS, pH 7.5, 150 mM NaCl, 1% NP-40) containing Protease Inhibitor Cocktail (Sigma, St. Louis, MO) was added and cells were detached with a cell scraper and transferred into a 15 mL tube. Cells were lysed at 4°C for 30 minutes with end-over-end rotation. The cell lysate was centrifuged at 4°C, 1000 x g, for 10 minutes and the supernatant collected for use in affinity chromatography.

*Affinity chromatography/Mass spectrometry*

Purified InlF-His_6_ protein was dialyzed into cold 0.1 M MOPS, pH 7.5, at greater than 1 mg/mL protein concentration. Affi-Gel 15 affinity support (Bio-Rad, Hercules, CA) was washed and prepared for aqueous coupling according to the manufacturer’s recommendations. The moist gel cake was added to the cold InlF-His_6_ protein solution and mixed together at 4°C with end-over-end rotation for 18 hours. The next day, the InlF-Affi-Gel 15 mixture was transferred to a column and washed with 0.1 M MOPS, pH 7.5, until no absorbance at A_280_ could be detected. The column was equilibrated with at least 4 column volumes of low NP-40 buffer (PBS, pH 7.5, 150 mM NaCl, 0.1% NP-40) at 4°C. Equal volumes of low NP-40 buffer and L2 cell lysate were mixed together and applied to the InlF-Affi-Gel 15 column and allowed to cycle over the column for 4 hours at 4°C. The InlF-Affi-Gel 15 column was washed with at least 4 column volumes of low NP-40 buffer. Bound proteins were eluted off the column by addition of 3 column volumes of elution buffer (PBS, pH 7.5, 1 M NaCl, 7 M urea). The elution was precipitated with trichloroacetic acid (TCA), dried, and submitted for mass spectrometry analysis at the Taplin Mass Spectrometry Facility (Harvard Medical School, Boston, MA). The TCA precipitate was digested with trypsin and subjected to a reverse phase HPLC capillary column for peptide separation. As peptides eluted they were subjected to electrospray ionization and then entered into an LTQ Velos ion-trap mass spectrometer (ThermoFisher, San Jose, CA). Peptides were detected, isolated, and fragmented to produce a tandem mass spectrum of specific fragment ions for each peptide. Peptide sequences were determined by matching protein databases with the acquired fragmentation pattern by the software program, Sequest (ThermoFisher, San Jose, CA) ([3](#_ENREF_3)). Spectral matches were manually determined and multiple identified peptides per protein were required.

*Immunoprecipitation studies*

bEnd.3 cells seeded into 10-cm diameter tissue culture dishes were transfected with either mCherry-vimentin ([4](#_ENREF_4)) (Addgene, #55157) or RFP control vector (Clontech) using GeneJuice (Millipore). At 20 hours post-transfection, cells were washed in PBS and lysed in Lysis Buffer (50 mM Tris-HCl, 150 mM NaCl, 1 mM EDTA, 1% Triton X-100, pH 7.4) supplemented with phenylmethylsulfonyl fluoride (PMSF), 5 mM NaF, 5 mM NaVO_4_, 10 µg/mL aprotinin, 10 µg/mL leupeptin and 1 µM pepstatin. Cell lysates were added to RFP-Trap beads (Chromotek, #rta-20; 20 µl packed volume) and incubated for 1 hour at 4^o^C following three washes in lysis buffer. Each sample was then incubated with 10 µg of purified InlF-His_6_ for 2 hours. InlF-His_6_ was purified from the culture supernatant of *L. monocytogenes* strain DH-L1899. DH-L1899 bacteria were grown in 500 mL of LB media supplemented with chloramphenicol (30 µg/mL) at 37^o^C for 18 hours. Bacteria were pelleted and the culture supernatant was filtered through a 0.22 μm filter, and concentrated using Ultra Centrifugal Filters (Amicon) prior to purification using a Ni^2+^-charged HiTrap Chelating HP Column (GE Healthcare). InlF-His_6_ fractions were assessed for purity via SDS-PAGE and select eluates were dialysed 16-18 hours in PBS using a 10K Slide-A-Lyzer cassette (Thermo). RFP-Trap beads were washed three times in lysis buffer and bound proteins were eluted in 40 µL of 2X SDS-PAGE loading buffer at 95^o^C for 10 minutes. Protein eluates were resolved by 12% SDS-PAGE, transferred to PVDF membrane (Bio-Rad), and probed using either mouse anti-His_6_-tag (Thermo, #MA1-21315; 1:1000) or rat anti-RFP (Chromotek, #5f8-100; 1:1000) antibodies. Membranes were subsequently stained with respective HRP-conjugated secondary antibodies and detected using either Luminata Classico substrate (Millipore) or SuperSignal West Femto Maximum Sensitivity Substrate (Thermo). Densitometry was performed using the Image J program (National Institutes of Health, Rockville, MD).

*Immunofluorescence and confocal microscopy studies*

bEnd.3 monolayers were grown on glass coverslips in six-well plates and cells were pre-incubated with 1 µg/ml cytochalasin D (Sigma) for 15 minutes before infection with wild-type *L. monocytogenes*, Δ*inlF*, Δ*inlAB* Δ*inlF*, or Δ*inlAB* Δ*inlF*/pAM-*inlF* strains at an MOI of 25:1. After bacterial infection for 2 hours, bEnd.3 cells were then washed with warm PBS and fixed with 3% (w/v) paraformaldehyde for 10 minutes at room temperature (25°C) and prepared for fluorescence microscopy([5](#_ENREF_5)). Fixed samples were washed 4 times with PBS and blocked with 3% (w/v) BSA in PBS before subsequent incubations with primary and secondary antibodies. Coverslips were first incubated with a chicken anti-vimentin antibody and a rabbit polyclonal antibody against *L. monocytogenes* (BD Difco). Coverslips were then rinsed with PBS and treated with Alexa Fluor® 647 conjugated donkey anti-rabbit IgG (Jackson ImmunoResearch, West Grove, PA) and Alexa Fluor® 555 conjugated goat anti-chicken IgG (Molecular Probes) together with DAPI for labeling nuclei. Specimens were mounted for microscopy using ProLong Diamond mounting medium (Life Technologies, Eugene, OR) and analyzed by fluorescence microscopy. Confocal images were acquired with a FluoView FV3000 microscope (Olympus) with a 40X oil immersion objective lens (NA: 1.2) and processed with FluoView software (Olympus). To quantify vimentin co-localization with *L. monocytogenes*, Δ*inlF*, Δ*inlAB* Δ*inlF*, or Δ*inlAB* Δ*inlF*/pAM-*inlF* bacteria, co-localization of surface vimentin with *L. monocytogenes* was assessed from 10-15 different confocal image fields examining >400 bacteria for each strain. Data were analyzed using NIH ImageJ software and three-dimensional models and orthogonal views were created.

*Statistical analysis*

Statistical analysis for gentamicin protection assays and immunofluorescence confocal microscopy was performed using the Student’s *t-*test (two-tailed, unpaired). Differences were considered significant at *P* < 0.05.

**Supplemental References**

1. Russo BC, Stamm LM, Raaben M, Kim CM, Kahoud E, Robinson LR, Bose S, Queiroz AL, Herrera BB, Baxt LA, Mor-Vaknin N, Fu Y, Molina G, Markovitz DM, Whelan SP, Goldberg MB. 2016. Intermediate filaments enable pathogen docking to trigger type 3 effector translocation. Nat Microbiol 1:16025.

2. Kirchner M, Higgins DE. 2008. Inhibition of ROCK activity allows InlF-mediated invasion and increased virulence of *Listeria monocytogenes*. Mol Microbiol 68:749-767.

3. Eng JK, McCormack AL, Yates JR. 1994. An approach to correlate tandem mass spectral data of peptides with amino acid sequences in a protein database. J Am Soc Mass Spectrom 5:976-989.

4. Rizzo MA, Davidson MW, Piston DW. 2009. Fluorescent protein tracking and detection: fluorescent protein structure and color variants. Cold Spring Harb Protoc 2009:pdb.top63.

5. Koudelka KJ, Destito G, Plummer EM, Trauger SA, Siuzdak G, Manchester M. 2009. Endothelial targeting of cowpea mosaic virus (CPMV) via surface vimentin. PLoS Pathog 5:e1000417.
